# Integrated analysis of long noncoding RNA interactions reveals the potential role in progression of human papillary thyroid cancer

**DOI:** 10.1002/cam4.1721

**Published:** 2018-10-14

**Authors:** Xin You, Yixin Zhao, Jing Sui, Xianbiao Shi, Yulu Sun, Jiahan Xu, Geyu Liang, Qingxiang Xu, Yongzhong Yao

**Affiliations:** ^1^ School of Medicine Southeast University Nanjing Jiangsu China; ^2^ Department of General Surgery Nanjing Drum Tower Hospital The Affiliated Hospital of Nanjing University Medical School Nanjing Jiangsu China; ^3^ Key Laboratory of Environmental Medicine Engineering Ministry of Education School of Public Health Southeast University Nanjing Jiangsu China

**Keywords:** competing endogenous RNAs network, long noncoding RNAs, papillary thyroid cancer, The Cancer Genome Atlas

## Abstract

Recent scientific evidence has suggested that long noncoding RNAs (lncRNAs) play an important part in tumorigenesis as an important member of competing endogenous RNAs (ceRNAs). Hundreds of RNA sequence data and relevant clinic information are freely accessible in The Cancer Genome Atlas (TCGA) datasets. However, the role of cancer‐related lncRNAs in papillary thyroid cancer (PTC) is not fully understood yet. In this study, we identified 461 RNA sequencing data from TCGA. Subsequently, 45 lncRNAs, 21 miRNAs, and 78 mRNAs were chosen to construct a ceRNA network of PTC. Then, we analyzed the correlation between these 45 PTC‐specific lncRNAs and clinic features and patient outcome. Thirty‐seven of these lncRNAs were found to be closely related to age, race, gender, lymph node metastasis, TNM staging system, and patient outcome. Additionally, three of them were linked to PTC patient overall survival. Eventually, we selected eight lncRNAs randomly and performed quantificational real‐time polymerase chain reaction (qRT‐PCR) in 28 newly diagnosed patients with PTC to verify the reliability of the above results. The results of qRT‐PCR are totally in agreement with the bioinformatics analysis. Additionally, it was found that HAND2‐AS1 was negatively related to tumor size (*P* < 0.05). The results were consistent with the bioinformatics analysis in TCGA. Taken together, we identified the differentially expressed lncRNAs and constructed a PTC ceRNA network. The study provides a new perspective and supplement for our understanding of lncRNAs in PTC development and reveals potential diagnostic and prognostic markers in PTC.

## 1 INTRODUCTION

Thyroid cancer remains the most prevalent malignant endocrine disorder with low mortality but increased incidence and recurrence. According to the United States cancer statistics, approximately 726 646 people lived with thyroid cancer in 2014, and there would be 56 870 new cases diagnosed as thyroid cancer in 2017.^1^ Moreover, by 2030, thyroid cancer was expected to be the fourth leading cancer diagnosis.^2^ Papillary thyroid cancer (PTC) is the most common pathological type, accounting for 80% of thyroid cancer. In the long run, the prognosis of PTC is excellent and the 5‐year survival rate could reach 98.2%; however, its recurrence rate is relatively high.^3^, ^4^ On the other hand, anaplastic thyroid cancer (ATC) is less frequent even if it has more aggressive biological behavior and worse prognosis. Recent studies showed that genetic and epigenetic alterations were involved in PTC pathogenesis and could be markers for prognosis, such as BRAF mutations,^5^, ^6^, ^7^ RAS mutations,^8^, ^9^ and RET rearrangements.^10^, ^11^

LncRNAs are described as transcripts with longer than 200 nucleotides without translation function. It plays an essential part in the regulation of gene transcription, post‐transcription, and epigenetic modulation.^12^, ^13^, ^14^ Amounting evidence indicate that lncRNAs are crucial in oncogenesis and tumor development.^15^, ^16^, ^17^ It was also found that plenty of lncRNAs was differentially expressed in thyroid cancer compared with normal adjacent tissues. These lncRNAs were expected to be correlated with diagnosis and prognosis of PTC.^18^

The competing endogenous RNA (ceRNA) hypothesis was first proposed about how messenger RNAs (mRNAs), transcribed pseudogenes, and lncRNAs connect to each other using microRNA response elements (MREs).^19^ Recently, ceRNA hypothesis was proved to be essential in physiologic and pathological conditions such as cancer.^20^

Thousands of lncRNAs were found aberrantly expressed in PTC samples compared with normal thyroid tissues using genomewide analysis.^21^ With further research, more and more lncRNAs were found to be closely related to the initiation and development of PTC. For instance, 218 aberrantly expressed lncRNAs were identified in genomewide expression screening, and two lncRNAs (XLOC‐051122 and XLOC‐006074) were found significantly related to lymphatic metastasis.^22^ Another study performed by Li et al^23^ described four lncRNAs signature (RP11‐536N17.1, RP11‐508M8.1, AC026150.8, and CTD‐2139B15.2) predicting PTC prognosis. Differentially expressed RNAs in 348 cases were identified in three PTC variants and were selected to construct the ceRNA network by Yanjing Zhao.^24^ However, the ceRNA network of PTC is still poorly investigated in a larger cohort, and the validation analysis needs to be performed. In this study, dysregulated RNAs (including lncRNAs, miRNAs, and mRNAs) were detected in different stages and lymph node status, and these RNAs were used to construct the ceRNA network of PTC. Next, quantificational real‐time polymerase chain reaction (qRT‐PCR) validation was performed to confirm the credibility of bioinformatics analysis results. GO terms and KEGG pathways were performed to identify the differentially expressed mRNAs. Our results indicate that ceRNA network plays an important part in the development of PTC, and the study also provides a novel perspective for the better understanding of lncRNA‐miRNA‐mRNA interactions in PTC initiation and development.

## 2 MATERIALS AND DESIGN

### 2.1 Patients and samples

Our study was granted approval by the ethics committee of Nanjing Drum Tower Hospital. Firstly, we collected 503 PTC cases and 59 normal thyroid cases from the The Cancer Genome Atlas (TCGA) database (https://cancergenome.nih.gov/). Then, we set the exclusion criteria as below: (a) pathologic diagnosis is not PTC; (b) suffering from other cancers except PTC; (c) patients samples without integrated clinical information. Overall, 461 PTC tumor cases and 55 normal thyroid cases were identified in the study. On the basis of the 7th American Joint Committee on Cancer (AJCC) TNM staging system, among these 461 patients with PTC, there were 256 patients with stage I PTC, 51 patients with stage II PTC, 104 patients with stage III PTC, and 50 patients with stage IV PTC. Additionally, there are 219 PTC cases with lymphatic metastasis and 242 PTC cases without lymphatic metastasis.

Additionally, 28 paired frozen PTC samples (thyroid tumor samples and adjacent normal thyroid samples) were obtained from Nanjing Drum Tower Hospital for qRT‐PCR. Tissues were immersed in RNAlater (GenStar BioSolutions, Beijing, China) immediately after surgical resection and stored at −80°C until use. Pathology reports and quality assessment reports were required to verify the collected tumor tissues and normal thyroid tissues. Demographic and clinical features of these patients were shown in Table 1. Informed consent agreements were signed by all patients in this study.

**Table 1:** Demographic and clinical features of 28 patients with PTC

Parameters	Cohort (n = 28) %
Age (mean ± SD)	39.8 ± 9.8
Gender	
Female	20 (71.4)
Male	8 (28.6)
Pathologic staging	
I‐II	22 (78.6)
III‐IV	6 (21.4)
Tumor size	
T1‐T2	17 (60.7)
T3‐T4	11 (39.3)
Lymph node	
N0	10 (35.7)
N1	18 (64.3)
Metastasis	
M0	28 (100)
M1	0 (0)

### 2.2 RNA sequence data analysis

**Figure 1:**
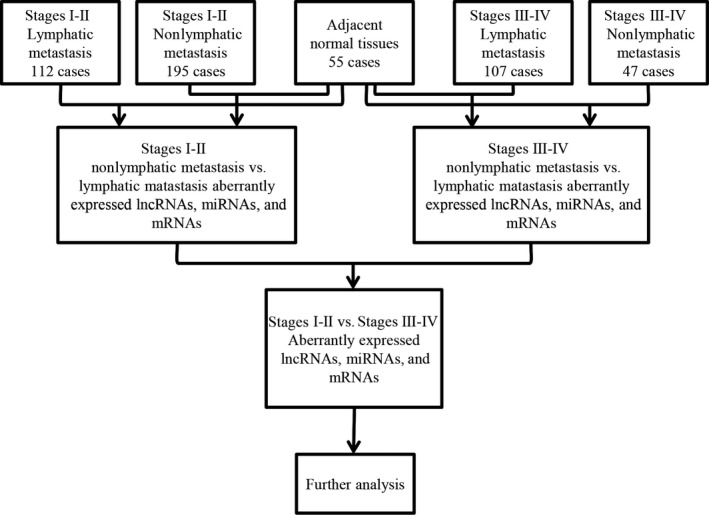
The flowchart of bioinformatics analysis

Firstly, we obtained all RNA expression profiles (level 3) of patients with PTC from the TCGA data portal (up to May 05, 2016). The raw data of lncRNAs and mRNAs were processed and normalized by TCGA RNASeqv2 system. The PTC level 3 miRNA data were normalized and provided by Illumina HiSeq 2000 microRNA sequencing platforms (Illumina Inc., Hayward, CA, USA). No further normalization was needed as all the RNA sequencing data have already been normalized already. Then, all samples collected in TCGA were divided into three groups to perform the differential analysis of lncRNAs, miRNAs, and mRNAs. Thus, all collected samples were grouped as: (a) PTC tumor samples and normal thyroid samples; (b) PTC samples with lymphatic metastasis and PTC samples without lymphatic metastasis; (c) PTC samples with stages I‐II and PTC samples with stages III‐IV. Four groups were listed as follows: stages I‐II with/without lymphatic metastasis and stages III‐IV with/without lymphatic metastasis, and these groups contained 112, 195, 107, and 47 cases, respectively. The intersected lncRNAs, miRNAs, and mRNAs were then chosen to construct the ceRNA network which shows the genetic regulation related to PTC. The bioinformatics analysis is illustrated in the flowchart (Figure 1).

### 2.3 Gene functional enrichment analysis

The intersected mRNAs were selected following the above methods and were analyzed to detect the potential biologic functions and pathways. The Database for Annotation, Visualization, and Integrated Discovery (DAVID) Bioinformatics resources (http://david.abcc.ncifcrf.gov/) were employed to analyze the potential functions and pathways. The biological functions and pathways were considered significant when an enrichment score of Gene Ontology (GO) and Kyoto Encyclopedia of Genes and Genomes (KEGG) analysis exceeds 1.5 (*P* < 0.05).

### 2.4 The ceRNA network in PTC

**Figure 2:**
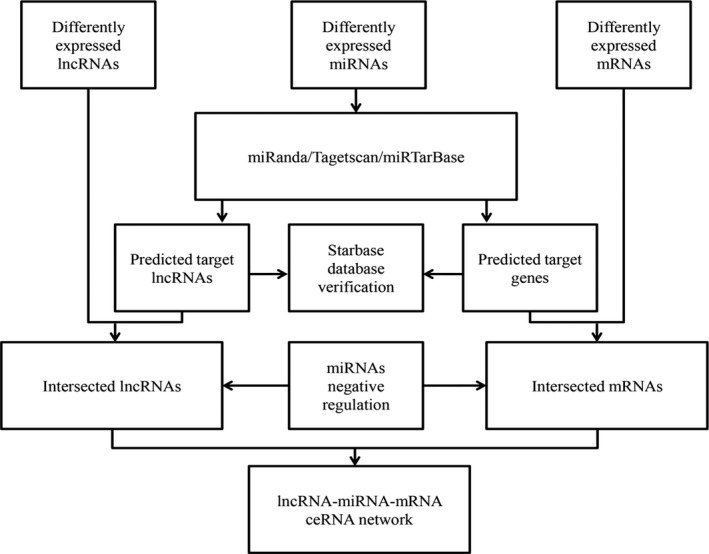
The flowchart of ceRNA network analysis

The interactions among lncRNAs, miRNAs, and mRNAs contribute to the post‐transcriptional regulation of mRNAs. The ceRNA network is built based on miRNA sponge^25^ in our study. This theory indicates that lncRNAs conjunct with miRNAs to regulate mRNAs expression. Furthermore, the miRNA‐lncRNA and miRNA‐mRNA relationships were collected in the starBase v2.0 database (http://starbase.sysu.edu.cn/),^26^ and only differentially expressed intersection lncRNAs, miRNAs, and mRNAs with fold change (FC) >2.0 or FC <0.5 and *P* < 0.05 were selected. Subsequently, miRanda tools (http://www.microrna.org/microrna/home.do), the miRNAs target prediction tool, were employed to detect the lncRNA‐miRNA interactions. Then, the mRNA‐miRNA interactions were analyzed by miRTarBase (http://mirtarbase.mbc.nctu.edu.tw/) and TargetScan (http://www.targetscan.org/). The summary results were identified to determine the specific interacted lncRNAs and mRNAs. Then, according to ceRNA theory, the negatively regulated lncRNA‐miRNA and mRNA‐miRNA pairs were analyzed in the ceRNA network in PTC.^27^ Finally, the ceRNA network was built using Cytoscape v3.0.^28^ The ceRNA network construction is illustrated in a flowchart (Figure 2).

### 2.5 Clinical features analysis and qRT‐PCR verification

Specific lncRNAs involved in ceRNA network and bioinformatics analysis were selected to assess the clinical characteristics including age, race, gender, lymphatic metastasis, TNM staging system, and patient outcome in TCGA. In addition, qRT‐PCR validation of eight randomly selected lncRNAs in 28 paired frozen tissues was performed to examine the credibility of the bioinformatics analysis results.

Following the manufacturer's protocol, total RNAs were extracted and purified using TRIzol reagent (Pufei, Shanghai, China). Then, concentration and purity of total RNA were detected by the spectrophotometer from BioTek (Shoreline, WA, USA). According to the manufacturer's protocol, a total of 1000 ng RNA was used to perform cDNA synthesis, and reverse transcription reaction was conducted using PrimeScript™ from Takara (Dalian, China). Candidate lncRNA expression was amplified with TAKARA SYBR^®^ Premix Ex Taq™ (Dalian, China) on an Applied Biosystems ViiA™ 7 System (Foster City, CA, USA) using specific primers in duplicate. All primers were designed and synthesized by Generay Biotech (Shanghai, China), and the sequences of selected lncRNAs primers were presented in Table 2. qPCR primers were determined to anneal at 60°C according to the instructions. The comparative CT method was used to measure the relative expression of candidate lncRNAs.^29^ β‐actin is used as the internal control in our experiment. FC values were shown as 2^−∆∆Ct^, where ∆∆Ct = (Ct_RNAs_ − Ct_β‐actin_)_tumor_ − (Ct_RNAs_ − Ct_β‐actin_)_adjacent normal samples_.

**Table 2:** The sequences of eight lncRNAs primers

	Forward primer (5′→3′)	Reverse primer (5′→3′)
β‐ACTIN	CTACCTCATGAAGATCCTCACCGA	TTCTCCTTAATGTCACGCACGATT
LOC100130238	CAAAACGAAACCCCTTACTGC	ATCCCCTAGATCAAGCCATGC
HAND2‐AS1	GGAGTCACAGGCAGTCGTAGA	GAAGGCACAGATCATTCATGG
MIR9‐3HG	CTCTGCCCTCCTACTTACGCT	CTGCTGGTCATCTGCATTCCT
LOC143666	CTCCCTGTGGTGCTTGAATGA	TCTACCGCTATCTACTACGAACTT
EGFEM1P	AATTGAGACACTGGAAGGTGAT	TTGAGTAGCGGTTGATTTGGT
LINC00284	AGGTTTCCCTCCTTGGCTTAC	TCACATCAGGTCCTTTGCTCC
TINCR	CCACTGTCATCTCCCCTCTTT	TCTCCCTCCCTATCTTCCATT
ABCC6P1	TACAGAAACTGCCAGGTCAAG	AGAAGACAGAGGAGCAGACAAA

### 2.6 Statistical analysis

The Student's *t* test was performed to detect the statistical significance for two groups, and ANOVA for multiple groups using the GraphPad Prism 6.0. All data are expressed as mean ± SD (standard deviation). The threshold of false discovery rate (FDR) was set as 0.001 to reduce the false positives in multiple tests in bioinformatics analysis, and the threshold of *P* value was set as 0.05 to evaluate the null hypothesis. Box plots were used to present the FC and clinical relevance of the specific lncRNAs in donor samples. The upper and lower boundaries of the boxplots are at 25th and 75th percentiles. The vertical lines below and above the box represent the minimum and the maximum. And the band inside the box is always the median.

## 3 RESULTS

### 3.1 PTC‐specific lncRNAs

**Figure 3:**
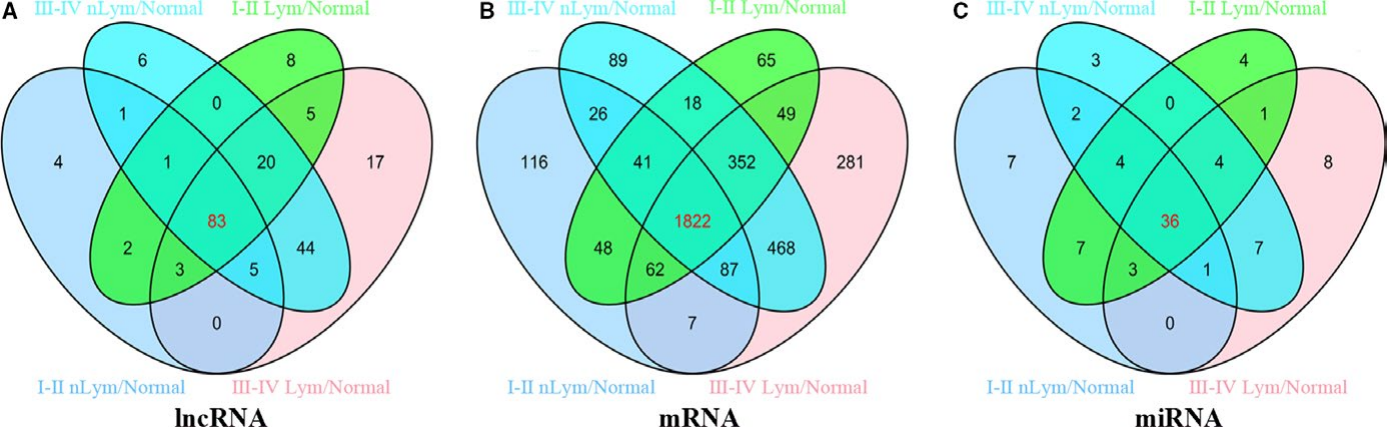
Venn diagram analysis of differentially expressed (A) lncRNAs, (B) mRNAs, and (C) miRNAs between I‐II Lym/Normal, I‐II nLym/Normal, III‐IV Lym/Normal, and III‐IV nLym/Normal. Lym, lymph node metastasis; nLym, nonlymph node metastasis; normal represents adjacent nontumor thyroid tissues

Totally, 1806 lncRNAs were identified from TCGA database (Data S1). We compared the expression of lncRNAs in 461 patients with PTC tumor tissues with adjacent normal tissues and identified 199 differentially expressed lncRNAs (FC > 2, *P* < 0.01). Further analysis was carried out between tumor tissues and adjacent normal thyroid tissues on tumor stages and lymph node status. For patients without lymphatic metastasis, there are 99 aberrant expression lncRNAs between stages I‐II PTC tumors samples and normal thyroid samples, and 122 aberrant expression lncRNAs between stages III‐IV PTC tumors samples and normal thyroid samples. For patients with lymphatic metastasis, 160 aberrant expression lncRNAs were identified between stages I‐II PTC tumors samples and normal thyroid samples, and 177 aberrant expression lncRNAs were identified between stages III‐IV PTC tumors samples and normal thyroid samples (Figure 3A). The intersected 83 specific intersected lncRNAs were selected initially for further analysis on ceRNA network to improve the credibility of bioinformatics analysis results (Table 3).

**Table 3:** Differentially expressed intersection lncRNAs between I‐II Lym/Normal, I‐II nLym/Normal, III‐IV Lym/Normal, and III‐IV nLym/Normal

lncRNAs	Gene ID	Regulation	Average FC
LOC400794	400794	Up	55.465
RPSAP52	204010	Up	15.625
TINCR	257000	Up	11.360
ABCC6P1	653190	Up	8.105
PRSS3P2	154754	Up	7.165
EGFEM1P	93 56	Up	6.810
NR2F1‐AS1	441094	Up	6.600
FLJ16779	100192386	Up	6.365
MIR31HG	554202	Up	6.095
MIR924HG	647946	Up	5.490
LINC00284	121838	Up	5.455
ABHD11‐AS1	171022	Up	5.190
FBLL1	345630	Up	4.650
CYP1B1‐AS1	285154	Up	4.585
LOC100126784	100126784	Up	4.585
FLJ23867	200058	Up	4.290
LINC00887	100131551	Up	4.130
YWHAEP7	284100	Up	4.060
SFTA1P	207107	Up	3.955
LOC93429	93429	Up	3.935
LOC441204	441204	Up	3.865
FER1L4	80307	Up	3.860
HCG22	285834	Up	3.595
GGT8P	645367	Up	3.465
FOXD2‐AS1	84793	Up	3.340
PP14571	100130449	Up	3.260
EGOT	100126791	Up	3.255
ABCC6P2	730013	Up	3.035
LBX2‐AS1	151534	Up	2.880
FCGR1CP	100132417	Up	2.760
SMIM10L2A	399668	Up	2.740
GGT3P	2679	Up	2.725
KRTAP5‐AS1	338651	Up	2.590
LINC00152	112597	Up	2.575
CYP2B7P	1556	Up	2.450
DLEU2	8847	Up	2.345
LOC285629	285629	Up	2.255
MBL1P	8512	Up	2.240
ESPNP	284729	Up	2.210
LINC01366	257358	Up	2.150
PLEKHA8P1	51054	Up	2.125
AADACP1	201651	Down	−11.976
LINC00473	90632	Down	−11.236
SLC26A4‐AS1	286002	Down	−10.582
TPTE2P1	646405	Down	−10.363
LOC100130238	100130238	Down	−10.152
FAM167A‐AS1	83656	Down	−7.143
LINC01139	339535	Down	−7.143
TDH	157739	Down	−5.128
HAND2‐AS1	79804	Down	−5.000
LINC00092	100188953	Down	−4.762
LINC01257	116437	Down	−4.444
VLDLR‐AS1	401491	Down	−4.348
LOC143666	143666	Down	−4.348
DPY19L2P4	442523	Down	−4.167
MIR9‐3HG	254559	Down	−4.082
LINC00982	440556	Down	−4.082
B3GALT5‐AS1	114041	Down	−3.846
LINC01550	388011	Down	−3.774
GOLGA8IP	283796	Down	−3.571
LINC00602	441177	Down	−3.509
MIR4697HG	283174	Down	−3.448
FAM181A‐AS1	283592	Down	−3.448
ANKRD20A8P	729171	Down	−3.390
ATP6V0E2‐AS1	401431	Down	−3.279
FAM95B1	100133036	Down	−3.125
LINC00652	29075	Down	−3.030
SNORD116‐4	100033416	Down	−3.030
ST7‐AS1	93653	Down	−2.817
LINC01140	339524	Down	−2.817
TERC	7012	Down	−2.778
PWAR5	8123	Down	−2.778
LINC01126	100129726	Down	−2.740
MIR22HG	84981	Down	−2.667
ZNF826P	664701	Down	−2.667
LRRC37A6P	387646	Down	−2.632
PGM5‐AS1	572558	Down	−2.632
LINC00261	140828	Down	−2.597
LINC00936	338758	Down	−2.597
FAR2P1	440905	Down	−2.597
AGPAT4‐IT1	79992	Down	−2.532
SNORD116‐20	100033431	Down	−2.532
GVINP1	387751	Down	−2.353

83 PTC‐specific lncRNAs for ceRNA network construction with absolute fold change (FC) >2.0, *P* < 0.05. I, II, III, and IV, TNM stages I, II, III, and IV. Lym, lymph node metastasis; nLM, nonlymph node metastasis; normal represents adjacent nontumor thyroid tissues.

### 3.2 Functional enrichment analysis

DAVID bioinformatics resources were employed to predict the biological functions and pathways of aberrantly expressed intersection mRNAs. A total of 18 633 mRNAs was identified from TCGA database, and 3531 mRNAs were aberrantly expressed in PTC tumor samples compared with normal thyroid samples (FC >2, *P* < 0.01). For patients without lymphatic metastasis, 2209 aberrant expression mRNAs were identified between stages I‐II PTC tumors samples and normal thyroid samples, and 2457 aberrant expression mRNAs were identified between stages III‐IV PTC tumors samples and normal thyroid samples. For patients with lymphatic metastasis, 2903 aberrant expression mRNAs were identified between stages I‐II PTC tumors samples and normal thyroid samples, and 3128 aberrant expression mRNAs were identified between stages III‐IV PTC tumors samples and normal thyroid samples. Finally, we identified 1822 mRNAs from the intersection of the four groups for further functional analysis.

**Figure 4:**
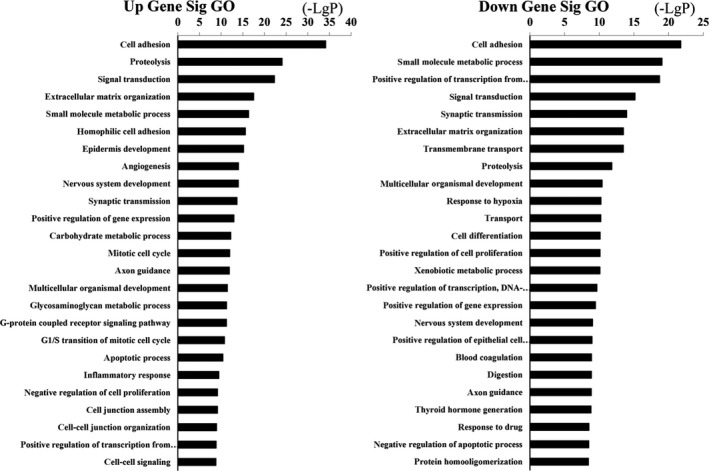
Top 25 enrichment of GO terms for differentially expressed intersection mRNAs (the bar plot shows the enrichment scores of the significant top 25 enrichment GO terms)

**Figure 5:**
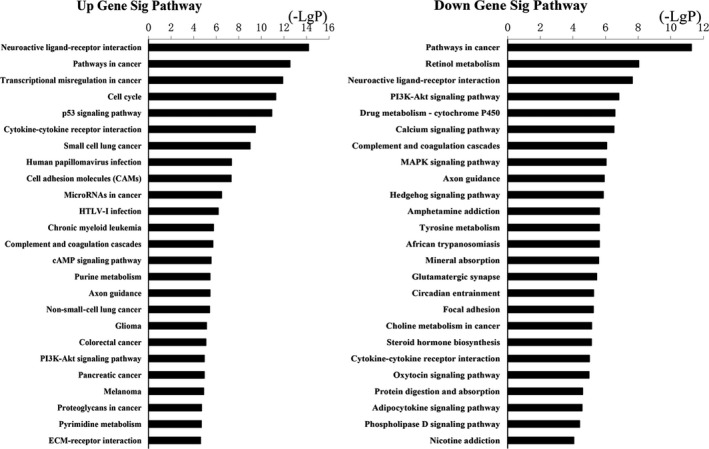
Top 25 enrichment of pathways for differentially expressed intersection mRNAs (the bar plot shows the enrichment scores of the significant enrichment pathways)

The upregulation and downregulation of these intersected mRNAs were further analyzed, respectively. GO analysis revealed 531 biological processes corresponding to these upregulated genes and 692 biological processes corresponding to these downregulated genes. Cell adhesion (GO:0007155) was the most enriched function for both upregulation and downregulation transcripts (Figure 4). KEGG pathway indicated 100 pathways matching with upregulated genes, and the top pathway was neuroactive ligand‐receptor interaction. Furthermore, pathway analysis showed 101 pathways matching with downregulated genes and the top pathway was pathways in cancer (Figure 5). Among these pathways, “MAPK signaling pathway” has already been demonstrated as the main cause of genetic alterations and PTC development. Targeting “MAPK signaling pathway” has shown an effective antitumor effect in preclinical studies and ongoing clinical trials.^30^

### 3.3 Prediction of miRNAs targets and construction of ceRNA network

A total of 1030 miRNAs were obtained from TCGA database to identify potential differentially expressed miRNAs. And it was found that 87 miRNAs were aberrantly expressed in PTC tumor samples compared with normal thyroid samples (FC > 2, *P* < 0.05). According to the comparisons of four groups described above, 36 miRNAs were selected from the intersections of 87 PTC‐associated miRNAs to build the ceRNA network. Consequently, miRNAs target prediction including lncRNAs and mRNAs were conducted following the instructions described above. Firstly, 83 intersected lncRNAs and 36 specific miRNAs were collected to analyze the potential relationship in PTC. The potential miRNA‐lncRNA interactions were predicted using StarBase v2.0 which could explore the candidate MREs. Finally, according to the analysis results, there were 29 specific miRNAs interacting with 54 specific lncRNAs (Table 4).

**Table 4:** miRNAs that may target PTC‐specific lncRNAs

lncRNAs	miRNAs
ABCC6P1	hsa‐miR‐214‐3p
ABCC6P2	hsa‐miR‐214‐3p
ABHD11‐AS1	hsa‐miR‐34a‐5p
AGPAT4‐IT1	hsa‐miR‐214‐3p
ANKRD20A8P	hsa‐miR‐221‐5p, hsa‐miR‐9‐5p
ATP6V0E2‐AS1	hsa‐miR‐204‐5p, hsa‐miR‐34a‐5p, hsa‐miR‐9‐5p
CDC14C	hsa‐miR‐9‐5p
CYP2B7P	hsa‐miR‐138‐5p, hsa‐miR‐214‐3p
DLEU2	hsa‐miR‐187‐3p, hsa‐miR‐199a‐3p
DPY19L2P4	hsa‐miR‐146b‐5p
EGFEM1P	hsa‐miR‐138‐5p, hsa‐miR‐181a‐5p, hsa‐miR‐181b‐5p, hsa‐miR‐214‐3p, hsa‐miR‐222‐5p, hsa‐miR‐363‐3p, hsa‐miR‐509‐3p
EGOT	hsa‐miR‐214‐3p
FAM181A‐AS1	hsa‐miR‐138‐5p, hsa‐miR‐514a‐3p
FAM95B1	hsa‐miR‐146b‐3p
FAR2P1	hsa‐miR‐138‐5p, hsa‐miR‐146b‐3p, hsa‐miR‐199a‐3p, hsa‐miR‐214‐3p, hsa‐miR‐221‐3p, hsa‐miR‐221‐5p, hsa‐miR‐222‐3p, hsa‐miR‐3065‐5p
FCGR1CP	hsa‐miR‐138‐5p
FER1L4	hsa‐miR‐138‐5p, hsa‐miR‐146b‐3p, hsa‐miR‐187‐3p, hsa‐miR‐34a‐5p, hsa‐miR‐7‐5p
FLJ16779	hsa‐miR‐138‐5p, hsa‐miR‐181a‐2‐3p, hsa‐miR‐187‐3p, hsa‐miR‐214‐3p, hsa‐miR‐221‐5p,
FLJ23867	hsa‐miR‐214‐3p
FOXD2‐AS1	hsa‐miR‐214‐3p
GGT3P	hsa‐miR‐138‐5p, hsa‐miR‐199a‐3p, hsa‐miR‐214‐3p
GGT8P	hsa‐miR‐214‐3p
GVINP1	hsa‐miR‐144‐5p, hsa‐miR‐146b‐3p, hsa‐miR‐181a‐5p, hsa‐miR‐181b‐5p, hsa‐miR‐199a‐3p, hsa‐miR‐204‐5p, hsa‐miR‐214‐3p, hsa‐miR‐3065‐5p, hsa‐miR‐34a‐5p, hsa‐miR‐508‐3p, hsa‐miR‐7‐5p, hsa‐miR‐9‐5p
HAND2‐AS1	hsa‐miR‐138‐5p, hsa‐miR‐144‐5p, hsa‐miR‐146b‐5p, hsa‐miR‐204‐5p, hsa‐miR‐509‐3p, hsa‐miR‐514a‐3p
KRTAP5‐AS1	hsa‐miR‐146b‐3p, hsa‐miR‐199b‐5p
LBX2‐AS1	hsa‐miR‐675‐3p
LINC00261	hsa‐miR‐138‐5p, hsa‐miR‐146b‐3p, hsa‐miR‐204‐5p, hsa‐miR‐222‐5p, hsa‐miR‐34a‐5p, hsa‐miR‐9‐5p
LINC00284	hsa‐miR‐9‐5p
LINC00652	hsa‐miR‐214‐3p
LINC00887	hsa‐miR‐138‐5p, hsa‐miR‐181b‐5p, hsa‐miR‐204‐5p
LINC00982	hsa‐miR‐146b‐3p, hsa‐miR‐34a‐5p, hsa‐miR‐9‐5p
LINC01140	hsa‐miR‐138‐5p, hsa‐miR‐146b‐3p, hsa‐miR‐181b‐5p, hsa‐miR‐204‐5p, hsa‐miR‐214‐3p
LINC01257	hsa‐miR‐34a‐5p
LINC01366	hsa‐miR‐34a‐5p
LINC01550	hsa‐miR‐675‐3p
LOC100126784	hsa‐miR‐1247‐5p, hsa‐miR‐34a‐5p, hsa‐miR‐451a, hsa‐miR‐7‐5p
LOC100130238	hsa‐miR‐34a‐5p
LOC143666	hsa‐miR‐34a‐5p
LOC285629	hsa‐miR‐146b‐3p, hsa‐miR‐31‐5p, hsa‐miR‐9‐5p
LOC441204	hsa‐miR‐138‐5p
LOC93429	hsa‐miR‐138‐5p
LRRC37A6P	hsa‐miR‐146b‐3p, hsa‐miR‐146b‐5p, hsa‐miR‐187‐3p, hsa‐miR‐199a‐3p, hsa‐miR‐221‐3p, hsa‐miR‐222‐5p, hsa‐miR‐508‐3p
MIR31HG	hsa‐miR‐214‐3p
MIR4697HG	hsa‐miR‐146b‐3p, hsa‐miR‐181a‐5p, hsa‐miR‐204‐5p, hsa‐miR‐221‐5p, hsa‐miR‐31‐5p, hsa‐miR‐34a‐5p, hsa‐miR‐486‐5p, hsa‐miR‐7‐5p
MIR9‐3HG	hsa‐miR‐146b‐3p, hsa‐miR‐214‐3p, hsa‐miR‐221‐5p, hsa‐miR‐31‐5p
NR2F1‐AS1	hsa‐miR‐199b‐5p, hsa‐miR‐204‐5p,
PLEKHA8P1	hsa‐miR‐204‐5p
PRSS3P2	hsa‐miR‐34a‐5p
PWAR5	hsa‐miR‐3065‐5p, hsa‐miR‐31‐5p
RPSAP52	hsa‐miR‐222‐5p
SMIM10L2A	hsa‐miR‐146b‐3p, hsa‐miR‐187‐3p, hsa‐miR‐214‐3p, hsa‐miR‐221‐5p, hsa‐miR‐34a‐5p, hsa‐miR‐9‐5p
TINCR	hsa‐miR‐1247‐5p, hsa‐miR‐214‐3p
TPTE2P1	hsa‐miR‐146b‐3p, hsa‐miR‐199b‐5p, hsa‐miR‐214‐3p, hsa‐miR‐3065‐5p, hsa‐miR‐7‐5p, hsa‐miR‐9‐5p
YWHAEP7	hsa‐miR‐199b‐5p

**Figure 6:**
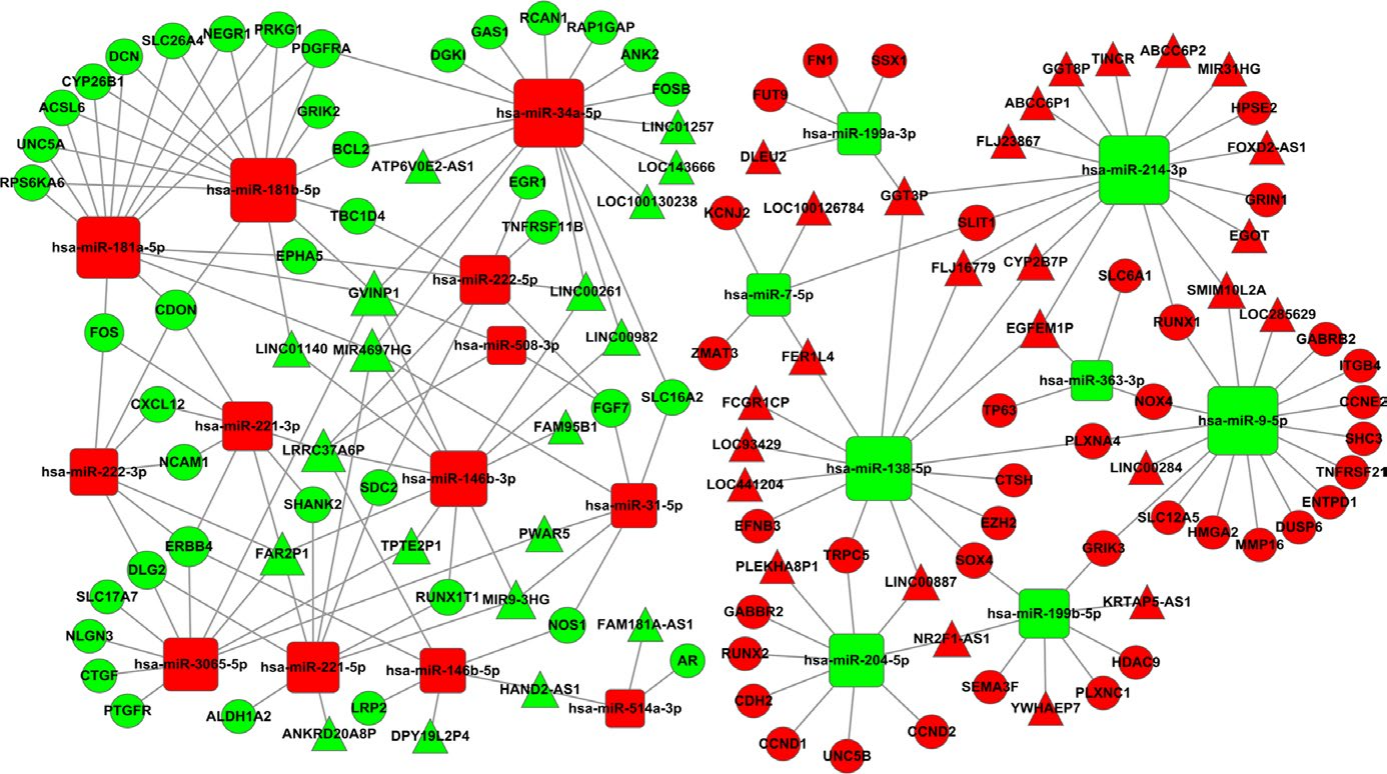
The lncRNA‐miRNA‐mRNA ceRNA network. Red diamonds represent upregulated miRNAs, red balls, upregulated mRNAs, and red cones, upregulated lncRNAs. Green diamonds represent downregulated miRNAs, green balls, downregulated mRNAs; green cones, downregulated lncRNAs

In the further analysis, the miRNA‐targeted mRNAs were predicted using TargetScan and miRTarBase. It was found that 30 miRNAs interacted with 130 mRNAs (Table 5). Then, we built a ceRNA network based on the above results (Tables 4 and 5). Cytoscape 3.0 was performed to depict the ceRNA network (Figure 6). The ceRNA network contains 45 lncRNAs, 21 miRNAs, and 78 mRNAs finally.

**Table 5:** miRNAs targeting PTC‐specific mRNAs

miRNAs	mRNAs
hsa‐miR‐1179	HDAC4, NCAM1, NRCAM, RUNX1, RUNX1T1, SGCD
hsa‐miR‐138‐1‐3p	CCND1, CCND2, EFNB3, ENPP1, FUT9, GPR83
hsa‐miR‐138‐5p	CTSH, EFNB3, ERBB4, EZH2, PLXNA4, PPARGC1A, RELN, SHANK2, SLC16A2, SLC17A7, SOX4, TCF7L1, TRPC5, UNC5A
hsa‐miR‐144‐3p	ERBB4, FGF7, FOSB, GABRB2, IRS1, ITPR1, LRP2, PLXNC1, RARB, SMAD9
hsa‐miR‐146b‐3p	CCND2, NRCAM, PPP1R1B, RUNX1T1, ERBB4, LRP2, MMP16, NOS1
hsa‐miR‐181a‐2‐3p	EFNB3, ERBB4, GLS2, HPSE2, OPRK1, SLC17A7
hsa‐miR‐181a‐5p	ACSL6, CDON, CYP26B1, DCN, EPHA5, FOS, NEGR1, PDGFRA, PRKG1, RPS6KA6, SHC3, SIPA1L2, SLC12A5, SLC26A4, TGFBR1, UNC5A
hsa‐miR‐181b‐5p	ACSL6, BCL2, CDON, CYP26B1, DCN, GRIK2, GRIK3, HEY2, NEGR1, PDE5A, PDGFRA, PLAU, PRKG1, RPS6KA6, SHC3, SIPA1L2, SLC26A4, TBC1D4, TGFBR1, UNC5A
hsa‐miR‐199a‐3p	DIO2, ERBB4, FN1, FUT9, RPS6KA6, SSX1
hsa‐miR‐199b‐5p	ERBB4, GRIK3, HDAC9, PLXNC1, PPARGC1A, RUNX1T1, SEMA3F, SGCD, SOX4
hsa‐miR‐204‐5p	AGPAT4, BCL2, CCND1, CCND2, CDH2, EPHA5, GABBR2, GPC3, PPARGC1A, RPS6KA5, RUNX2, SGCD, SLC22A3, TRPC5, UNC5B
hsa‐miR‐214‐3p	BMP8A, FOSB, GRIN1, HPSE2, KCNJ13, NOS1, NTN1, OPRK1, PPARGC1A, PTCH2, RUNX1, RUNX1T1, SEMA3D, SGCD, SLIT1, SV2B
hsa‐miR‐221‐3p	CDON, CXCL12, ERBB4, FOS, NCAM1, PLXNC1, SHANK2
hsa‐miR‐221‐5p	ADRA1B, ALDH1A2, CCND1, DLG2, HRH1, PLCD3, PPP1R1B, RUNX1T1, SDC2, SHANK2, SLC6A1, TP63
hsa‐miR‐222‐3p	CDON, CXCL12, DLG2, ERBB4, FOS, NCAM1
hsa‐miR‐222‐5p	CD44, EGR1, EPHA5, FGF7, SDC2, TBC1D4, TNFRSF11B
hsa‐miR‐3065‐5p	CTGF, DLG2, ERBB4, ETV5, FUT9, NLGN3, PTGFR, SLC17A7, SLIT1, SOX4
hsa‐miR‐31‐3p	CYP1B1
hsa‐miR‐31‐5p	CACNG4, FGF7, IL1RAP, NOS1, PLXNA4, RAPGEF5, SLC16A2
hsa‐miR‐34a‐5p	ANK2, BCL2, CCNE2, DGKI, F2RL2, FOSB, FUT9, GABBR2, GAS1, MET, PDGFRA, RAP1GAP, RCAN1, SLC16A2, SLC6A1, SOX4
hsa‐miR‐363‐3p	NOX4, SLC6A1, STEAP2, TP63
hsa‐miR‐375	SLC16A2
hsa‐miR‐486‐5p	EPHA3, GABRB3, SLC12A5, SRF
hsa‐miR‐508‐3p	FGF7, HMGA2, RAPGEF5
hsa‐miR‐509‐3p	ZMAT3
hsa‐miR‐514a‐3p	AR
hsa‐miR‐551b‐3p	ERBB4
hsa‐miR‐7‐2‐3p	BCL2, ETV5, GABBR2, GAS1, HDAC9, PDE5A, PPP1R1A, SHANK2
hsa‐miR‐7‐5p	CYTH3, EPHA3, IRS1, KCNJ2, SHANK2, SLIT1, WASF3, ZMAT3
hsa‐miR‐9‐5p	ANK2, CCNE2, CNTFR, DIO2, DUSP6, ENTPD1, GABRB2, GRIK3, HMGA2, ID4, ITGB4, LIFR, MMP16, NOX4, PLXNA4, RPS6KA6, RUNX1, RUNX1T1, SDC2, SGCD, SHANK2, SHC3, SLC12A5, SLC39A14, TBC1D4, TNFRSF21

### 3.4 The association between clinical characteristics and PTC‐specific lncRNAs

In the following analysis, the 45 specific lncRNAs identified in ceRNA network were evaluated to detect their clinical relevance, such as age, gender, race, lymphatic metastasis, TNM staging system, and patient outcome in TCGA database. It was found that a total of 37 specific lncRNAs expressed differentially correlated with PTC clinical features (*P* < 0.05, Table 6). Seven lncRNAs (ATP6V0E2‐AS1, FOXD2‐AS1, LINC01257, EGFEM1P, MIR9‐3HG, MIR31HG, and FER1L4) were differentially expressed in age, 4 lncRNAs (YWHAEP7, LINC00887, MIR4697HG, and MIR9‐3HG) were differentially expressed in race, 1 lncRNA (LINC00887) was differentially expressed in gender, 30 lncRNAs (ATP6V0E2‐AS1, LINC00982, NR2F1‐AS1, LOC100126784, DPY19L2P4, FLJ23867, TINCR, ANKRD20A8P, EGOT, FAM95B1, LOC143666, MIR4697HG, LRRC37A6P, LOC100130238, MIR31HG, TPTE2P1, FAR2P1, LINC01257, FAM181A‐AS1, FLJ16779, EGFEM1P, LOC93429, GGT8P, FER1L4, GGT3P, ABCC6P1, LINC00284, KRTAP5‐AS1, FOXD2‐AS1, and HAND2‐AS1) were differently expressed in lymphatic metastasis, 31 lncRNAs (LINC00982, FLJ23867, ABCC6P1, FAM181A‐AS1, FOXD2‐AS1, NR2F1‐AS1, EGOT, TINCR, LRRC37A6P, LOC100126784, TPTE2P1, LOC100130238, FLJ16779, DPY19L2P4, GGT3P, MIR4697HG, MIR9‐3HG, ANKRD20A8P, SMIM10L2A, GGT8P, FAM95B1, KRTAP5‐AS1, LOC441204, GVINP1, LINC00887, MIR31HG, HAND2‐AS1, EGFEM1P, LOC93429, ATP6V0E2‐AS1, and LINC01257) were aberrantly expressed in different tumor sizes, and six lncRNAs (PLEKHA8P1, LOC100130238, GGT3P, MIR9‐3HG, LINC00284, and EGFEM1P) were differentially expressed in prognosis.

**Table 6:** The correlations between PTC‐specific lncRNAs from ceRNA network and clinical features

Comparisons	Upregulation	Downregulation
Age (≥45 vs <45 years old)	ATP6V0E2‐AS1, FOXD2‐AS1, LINC01257	EGFEM1P, MIR9‐3HG, MIR31HG, FER1L4
Race (white vs Asian)	YWHAEP7, LINC00887	MIR4697HG, MIR9‐3HG
Gender (male vs female)	LINC00887	
Lymphatic metastasis (yes vs no)	NR2F1‐AS1, LOC100126784, FLJ23867, TINCR, EGOT, MIR31HG, FLJ16779, EGFEM1P, LOC93429, GGT8P, FER1L4, GGT3P, ABCC6P1, LINC00284, KRTAP5‐AS1, FOXD2‐AS1	ATP6V0E2‐AS1, LINC00982, DPY19L2P4, ANKRD20A8P, FAM95B1, LOC143666, MIR4697HG, LRRC37A6P, LOC100130238, TPTE2P1, FAR2P1, LINC01257, FAM181A‐AS1, HAND2‐AS1
TNM staging system (T3 + T4 vs T1 + T2)	FLJ23867, ABCC6P1, FOXD2‐AS1, NR2F1‐AS1, EGOT, TINCR, LOC100126784, FLJ16779, GGT3P, GGT8P, KRTAP5‐AS1, LOC441204, LINC00887, MIR31HG, EGFEM1P, LOC93429	LINC00982, FAM181A‐AS1, LRRC37A6P, TPTE2P1, LOC100130238, DPY19L2P4, MIR4697HG, MIR9‐3HG, ANKRD20A8P, SMIM10L2A, FAM95B1, GVINP1, HAND2‐AS1, ATP6V0E2‐AS1, LINC01257
Patient outcome (dead vs alive)	LOC100130238	PLEKHA8P1, GGT3P, MIR9‐3HG, LINC00284, EGFEM1P

**Figure 7:**
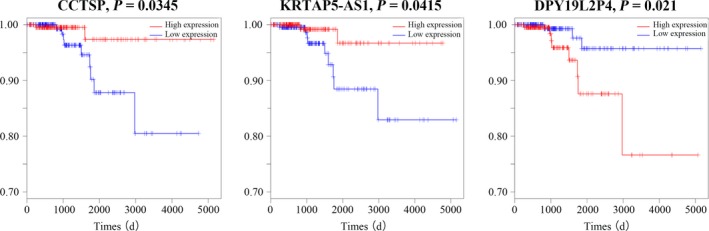
Kaplan–Meier survival curves for three lncRNAs associated with OS. Horizontal axis, OS time, days; vertical axis, survival function

The univariate Cox proportional hazards regression model was performed to detect the prognostic value of 45 specific lncRNAs. We found that three specific lncRNAs closely related with PTC patient overall survival (OS, log‐rank *P* < 0.05). There are two lncRNAs (GGT3P and KRTAP5‐AS1) positively relating to OS, and only one lncRNA (DPY19L2P4) negatively relating to OS (Figure 7).

### 3.5 qRT‐PCR validation

**Figure 8:**
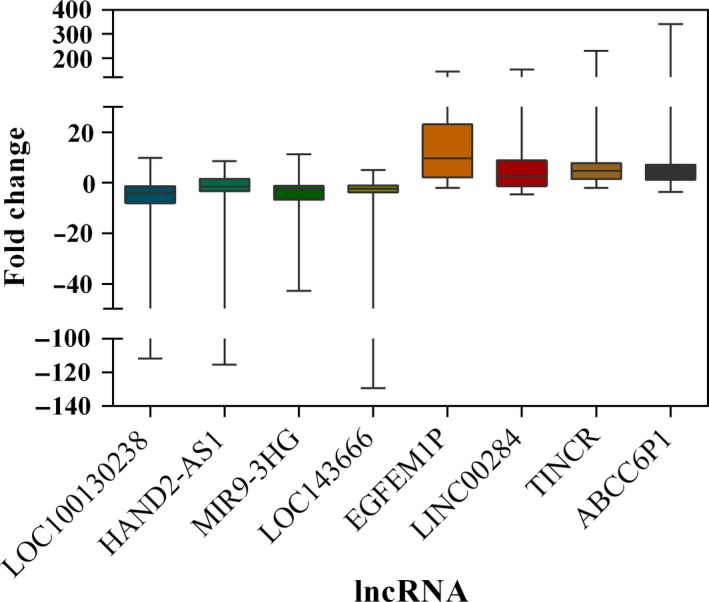
Box plot showing the median and quartiles of specific lncRNAs in donor samples

**Figure 9:**
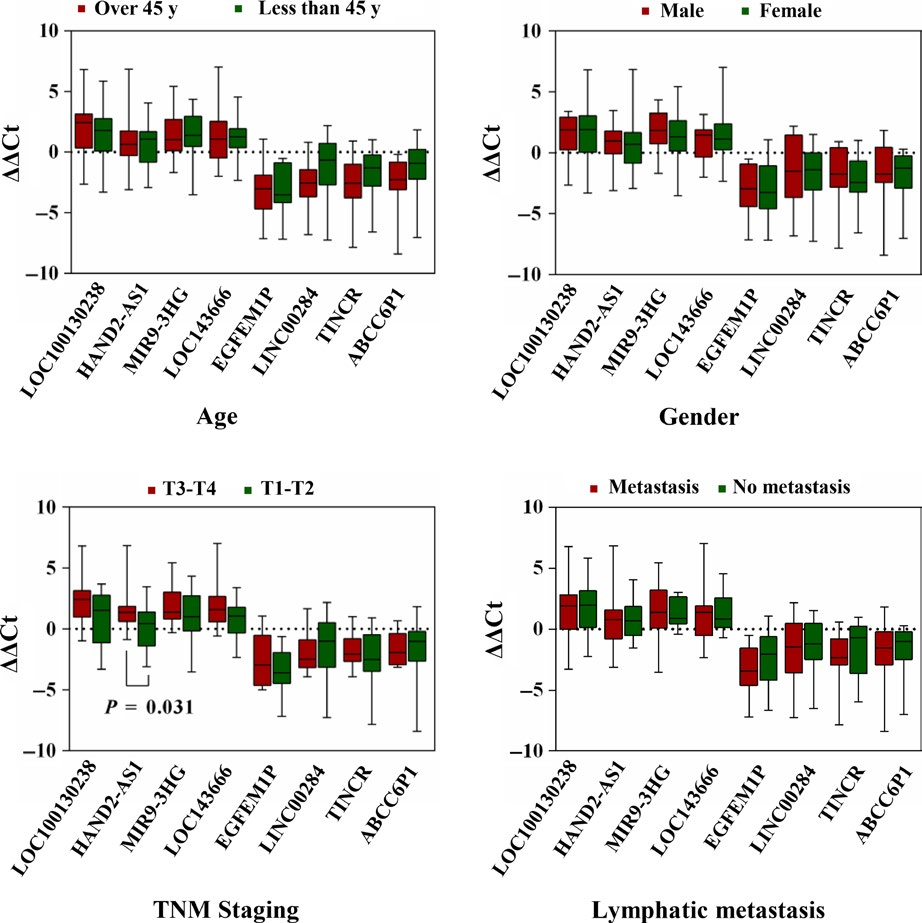
Box plot showing the association between the ΔΔCt of LOC100130238, HAND2‐AS1, MIR9‐3HG, LOC143666, EGFEM1P, LINC00284, TINCR and ABCC6P1 and clinicopathological characteristics in 28 PTC

Evaluation of randomly selected eight lncRNAs (LOC100130238, HAND2‐AS1, MIR9‐3HG, LOC143666, EGFEM1P, LINC00284, TINCR, and ABCC6P1) was performed to prove the reliability of the above bioinformatics analysis (Table 7). Firstly, the expression status of the eight specific lncRNAs was detected in 28 newly diagnosed patients with PTC using qRT‐PCR (Data S2). Compared with paired normal thyroid tissues, four lncRNAs (LOC100130238, HAND2‐AS1, MIR9‐3HG, and LOC143666) were downregulated in PTC tumor tissues, whereas the remaining four lncRNAs (EGFEM1P, LINC00284, TINCR, and ABCC6P1) were upregulated in PTC tumor tissues (Figure 8). All the eight lncRNAs were aberrantly expressed with the same trend and reached statistical significance (*P* < 0.05). Thus, the results were coherent with the above bioinformatics analysis. Subsequently, we performed the analysis of these eight lncRNAs and clinic features, and we found that HAND2‐AS1 was significantly related to tumor size (Figure 9, *P* < 0.05). The result was identical with the analysis in TCGA database. Thus, our bioinformatics analysis is convincing based on the evaluation results.

**Table 7:** Randomly selected lncRNAs with absolute fold change >2.0, *P* < 0.05

Names (lncRNAs)	Gene ID	Regulation	Average FC
LOC100130238	100130238	Down	9.69
HAND2‐AS1	79804	Down	4.82
MIR9‐3HG	254559	Down	4.44
LOC143666	143666	Down	4.35
EGFEM1P	93556	Up	6.94
LINC00284	121838	UP	5.05
TINCR	257000	Up	10.84
ABCC6P1	653190	Up	7.69

## 4 DISCUSSION

Thyroid cancer is a common malignant endocrine disorder worldwide with increasing incidence in last few decades. According to the National Cancer Center, PTC is the most frequently diagnosed cancer among Chinese women before 30 years old.^31^ We cannot overlook the rapid rising in PTC, although the use of ultrasonography and fine needle aspiration in clinical may lead to overdiagnosis and overtreatment of PTC. In general, PTC has a relatively favorable prognosis; however, there are still about 5%‐10% of patients suffering from an aggressive form of PTC.^32^ Under such circumstances, thyroid cancer medicine is facing a huge challenge nowadays since concerns may cause overtreatment of PTC. Conventionally, risk evaluation for PTC is only based on clinicopathologic factors, such as age and lymphatic metastasis, which are definitely insufficient to accurately distinguish high‐risk patients from low‐risk patients. Thus, it is urgent to explore the underlying genetic background of PTC in order to identify the aggressive PTC and avoid overtreatment clinically. Research concerning the genetic regulatory mechanism in thyroid cancer has shown sustained development.

It is evident that aberrant expression of lncRNAs is closely related to oncogenesis in various cancer types.^33^ It is notable that lncRNAs are crucial in the genetic regulation and are responsible for cellular hemostasis.^34^, ^35^ Moreover, many studies showed that lncRNAs play an important part in tumorigenesis and are regulated by tumor suppressors or oncogenes transcriptionally. However, there are only a few studies focusing on lncRNAs in PTC using whole genome and transcriptome sequencing technologies,^18^, ^36^, ^37^ which demonstrated that dysregulated lncRNAs are essential in PTC carcinogenesis and may serve as diagnostic, prognostic biomarkers, and even therapeutic targets. Genomic analysis of PTC was firstly conducted using microarray in 2015 by Lan Xiabin et al,^21^ which identified thousands of differentially expressed lncRNAs and mRNAs in PTC tissues compared with normal thyroid cancer. Additionally, genomewide expression screening in 12 paired PTC tumor tissues and normal thyroid tissues identified 218 differentially expressed lncRNAs in PTC, and two lncRNAs (XLOC‐051122 and XLOC‐006074) were found closely related to lymphatic metastasis.^22^ Du et al^38^ have performed a genomewide analysis in 18 PTC patients’ tissues and 4 healthy donors’ tissues, and build the coexpression network of lncRNA–mRNA. By now, much is unknown about lncRNAs and mRNAs, or lncRNAs and miRNAs in PTC based on ceRNA hypothesis. The ceRNA network in cancer research has drawn increasing attention as it was proposed in 2011.^19^ It presented a hypothesis about how ceRNAs regulated other RNA transcripts by competing for miRNAs. It could be seen that ceRNA network has been built in many cancer types, such as colorectal cancer^39^ and lung cancer.^40^ These results indicate that lncRNAs prove to be significant in tumorigenesis.

TCGA is the largest cancer genetic information database to study the genomic profiles of PTC. The ceRNA (lncRNA‐miRNA‐mRNA) network of PTC was once constructed based on the 348 PTC samples in TCGA.^24^ In this study, dysregulated RNAs were identified in three PTC variants, including classical PTC, follicular PTC, and tall‐cell PTC. However, the study missed part of the data in TCGA and lacked laboratory‐based studies to validate the bioinformatics results. We aimed to identify the specific lncRNAs with diagnostic and prognostic roles in the study. Firstly, we retrieved the lncRNAs, miRNAs, and mRNAs profiles correlated with PTC stage and lymphatic metastasis in 461 PTC cases from TCGA database. Subsequently, the ceRNA network was built to predict the genetic interactions among the specific lncRNAs, miRNAs, and mRNAs. Next, we selected the intersected lncRNAs, miRNAs, and mRNAs in ceRNA network to assess their values in clinical relevance and prognosis based on the RNA sequencing data of 461 PTC samples and 55 normal samples. To validate the analysis results, the expression level of eight randomly selected lncRNAs (LOC100130238, HAND2‐AS1, MIR9‐3HG, LOC143666, EGFEM1P, LINC00284, TINCR, and ABCC6P1) was detected in 28 newly diagnosed tumor tissues and paired normal tissues in PTC patients using qRT‐PCR. The evaluation displayed the consistent trend of up‐ and downregulation of selected lncRNAs with the expression level in TCGA. Eventually, the clinical information of donor samples was collected and compared with the data of TCGA. Similarly, the clinical relevance of lncRNAs was consistent with that of TCGA.

We classified PTC patients in TCGA into four groups based on TNM staging and lymph node status. Then, we compared the RNA sequence data of tumor tissues with that of normal thyroid tissues. The intersected mRNAs were finally decided accordingly. GO enrichment analysis and KEGG pathway analysis of intersected mRNAs were performed to reveal the potential mRNA functions. The top 25 GO enrichment terms indicated metastasis‐associated functions, such as cell adhesion, proteolysis, and extracellular matrix organization. As for KEGG pathway analysis, top 25 pathways of intersected mRNAs included many cancer‐specific pathways, such as MAPK, PI3K/Akt, and p53 signaling pathway. The results were consistent with the integrated genomic analysis in TCGA which demonstrated in detail about the importance of somatic genetic mutations, such as BRAF and RAS mutation, in the MAPK and PI3K pathways in PTC.^41^ Many studies have shown that BRAF gene mutations increased the MAPK signaling pathway activation which could promote the initiation and development of PTC.^42^, ^43^, ^44^ Generally, BRAF gene mutations are linked to larger tumor size, multifocality, extrathyroid extension, and lymphatic metastasis and predict poor prognosis.^7^, ^45^, ^46^ It was reported that AZD6244, a MAPK pathway inhibitor, was strongly promising to treat thyroid cancer.^47^ As the second most common gene mutation in thyroid cancer, RAS mutation mainly activates PI3K/Akt pathway.^48^, ^49^ Moreover, many drugs, such as temsirolimus and everolimus, targeting PI3K/Akt have been investigated in phases I to III clinical trials and proven to be effective in treating thyroid cancer.^50^ In addition, abnormal p53 activation is closely related to the development of thyroid cancer.^51^, ^52^ In conclusion, the functional analysis was closely related to the development of PTC, which was also coherent with the classification standards.

A great number of studies have presented that lncRNAs may function as ceRNAs and play an essential role in regulating gene expression.^53^, ^54^, ^55^, ^56^ For instance, lncRNA H19 and HULC may act through a ceRNAs manner by regulating let‐7a/let‐7b and miR‐372/miR‐373 which play an essential part in cholangiocarcinoma development.^57^ We constructed ceRNA network of PTC with aberrantly expressed lncRNAs, miRNAs, and mRNAs in TCGA. Moreover, it was also found some cancer‐specific lncRNAs, such as LINC01140, LINC00261, ABHD11‐AS1, and TINCR,^58^, ^59^, ^60^, ^61^ served as important biomarkers in other different cancer types as well. We also found certain lncRNAs in ceRNAs played important roles in carcinogeneses, such as FOXD2‐AS, DLEU2, FER1L4, HAND2‐AS1, and TPTE2P1.^62^, ^63^, ^64^, ^65^, ^66^ Next, we analyzed the biologic functions and pathways of identified mRNAs in ceRNA network. The results revealed many important functions and pathways, including MAPK, PI3K/Akt, and p53 signaling pathway.

We analyzed the correlation of 45 PTC‐specific lncRNAs and clinic features, including age, race, gender, lymph node metastasis, TNM staging system, and patient outcome. The result showed that 37 specific lncRNAs were correlated with the above clinic characteristics. Eleven of these 37 lncRNAs have been reported to be closely related to various cancer types. For instance, lncRNA MIR31HG might function as an endogenous “sponge” for competing miR‐193b binding to regulate gene expression and promote tumor progression in pancreatic ductal adenocarcinoma.^67^ So far, there are few reports on the clinical relevance of these specific lncRNAs and PTC. More laboratory‐based work needs to be done to verify the interaction of lncRNAs, miRNAs, and mRNAs in PTC. Eventually, we also analyzed the association between these 45 lncRNAs with patient OS. It was found that six lncRNAs (LOC100130238, PLEKHA8P1, GGT3P, MIR9‐3HG, LINC00284, and EGFEM1P) were linked to PTC OS.

Subsequently, qRT‐PCR validation of 8 lncRNAs in 28 paired samples was performed to assess the credibility and accuracy of the bioinformatics results. The results of qRT‐PCR were consistent with the expression data in TCGA. Next, we assessed the clinical relevance of these eight lncRNAs based on our collected clinical information. The results suggested that HAND2‐AS1 was closely related to tumor size, which is in coherence with the results of TCGA analysis. Hence, the bioinformatics analysis results of our study are credible and convincing.

Our study intended to identify the specific lncRNAs in PTC and investigate their genetic regulation by constructing the ceRNA network. Moreover, the expression status and clinical relevance of identified lncRNAs from TCGA database were assessed and compared with the data of donor samples. Finally, the credibility of bioinformatics analysis results was demonstrated and confirmed by qRT‐PCR. Taken together, our results suggested that lncRNA‐related ceRNA network might play an important role in the initiation and development of PTC. We also hope that our study can inspire researchers in this field to carry out further work.
